# Determining Microbial Roles in Ecosystem Function: Redefining Microbial Food Webs and Transcending Kingdom Barriers

**DOI:** 10.1128/mSystems.00153-19

**Published:** 2019-06-04

**Authors:** Kim M. Handley

**Affiliations:** a Genomics Aotearoa and School of Biological Sciences, University of Auckland, Auckland, New Zealand

**Keywords:** biogeochemical cycles, ecosystem function, functional genomics, microbial eukaryotes, microbial food webs

## Abstract

Microorganisms can have a profound and varying effect on the chemical character of environments and, thereby, ecological health. Their capacity to consume or transform contaminants leads to contrasting outcomes, such as the dissipation of nutrient pollution via denitrification, the breakdown of spilled oil, or eutrophication via primary producer overgrowth. Recovering the genomes of organisms directly from the environment is useful to gain insights into resource usage, interspecies collaborations (producers and consumers), and trait acquisition. Microbial data can also be considered alongside the broader biological character of an environment through the co-recovery of eukaryotic DNA. The contributions of individual microorganisms (bacteria, archaea, and protists) to snapshots of ecosystem processes can be determined by integrating genomics with functional methods. This combined approach enables a detailed understanding of how microbial communities drive biogeochemical cycles, and although currently limited by scale, key attributes can be effectively extrapolated with lower-resolution methods to determine wider ecological relevance.

## Abstract (precis)

Microorganisms can have a profound and varying effect on the chemical character of environments and, thereby, ecological health. Their capacity to consume or transform contaminants leads to contrasting outcomes, such as the dissipation of nutrient pollution via denitrification, the breakdown of spilled oil, or eutrophication via primary producer overgrowth.

## PERSPECTIVE

## FROM INFERRED LIFESTYLES TO MICROBIAL FOOD WEBS AND ECOSYSTEM FUNCTION

One of the obvious attractions of environmental genomics (and recovering metagenome-assembled genomes [MAGs]) is that it provides valuable insights into the lifestyles of uncultivated microorganisms and mechanisms of trait acquisition (e.g., plasmid-acquired function and chromosomal integration) (1). The recovery of environmental genomes also establishes a foundation from which microbial interactions can be inferred, based on the collective metabolic traits of communities (which illuminate potential producer-consumer relationships) (2), adaptations to symbiotic versus free-living lifestyles (3), and the genetic deficiencies of individuals. Recent explorations of environmental genomes illustrate that genome reduction and auxotrophy is rife (4, 5). Loss of genes required for the biosynthesis of essential amino acids, for example, explains why many microorganisms have eluded traditional mainstream cultivation efforts. Mounting genomic evidence suggests that the acquisition of exogenous metabolites, leaked from other organisms, is a common survival strategy, one that is predicted by the Black Queen Hypothesis to reflect a noncompetitive relationship between ecologically critical leaky helper organisms and dependent auxotrophs (6).

**FIG 1:**
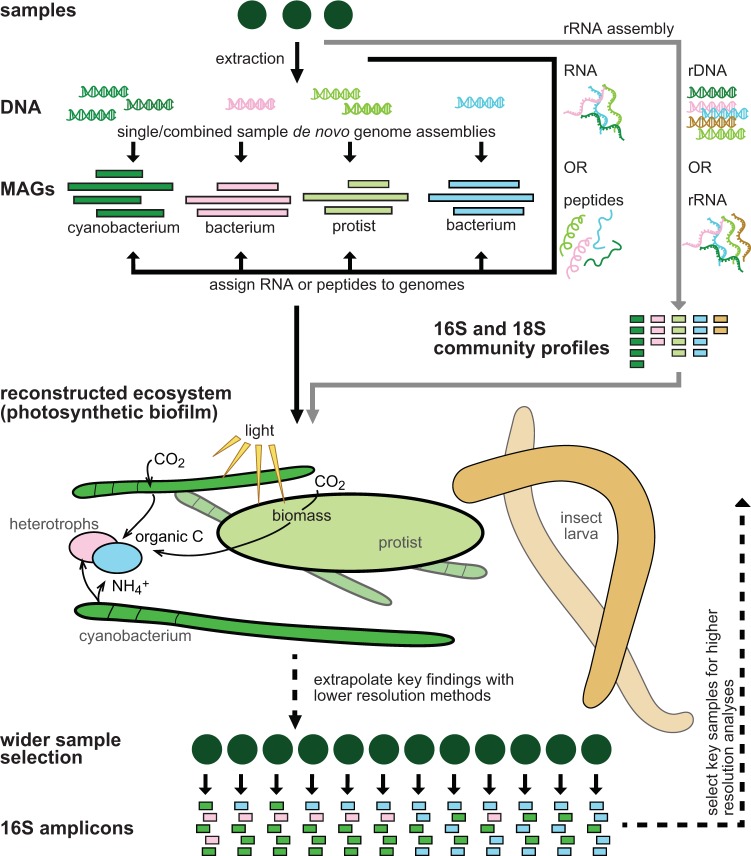
Schematic illustrating ecosystem reconstruction using functional genomics.

Understanding the metabolic capacity of organisms can reveal the types of resources (both organic and inorganic chemical species) that are important for sustaining life within an environment. For example, in addition to leaked metabolites (5), environmental genomic data indicate that inorganic carbon, reduced sulfur, nitrate, and hydrogen are important resources in the terrestrial subsurface (1). By identifying the likely recipients and producers of these goods, visual representations of microbial interactions, or food webs, can be constructed and microbial contributions to ecosystem functioning evaluated (Fig. 1). To this end, metabolic potentials derived from estuarine sediments have been used to graphically illustrate the diversity of organisms involved in breaking down complex organic carbon, and show how these organisms underpin important biogeochemical cycles (7). Results provide a useful guide to microbially driven estuarine processes. In comparison, my colleagues and I have used metagenomics and metaproteomics (metaproteogenomics) to identify key microbial interactions that occur when an alluvial aquifer is perturbed by excess organic carbon; schematic depictions show a dysbiotic community keenly focused on exchanging carbon, nitrogen, and sulfur species between trophic levels (2).

Complex microbial processes or interactions can be resolved using integrated approaches, such as functional genomics (which combines information from DNA, RNA, and proteins, e.g., genomics, transcriptomics, and/or proteomics), alongside host-related or geochemical data (8, 9). Geochemical data are particularly valuable for determining major metabolic end products. However, many nuanced biological processes, such as those producing and consuming intermediate metabolites, are better elucidated by functional genomics (e.g., where no intermediate products are detectable). This applies also to cryptic cycles, where metabolic products are recycled at fine spatial scales across sharp redox gradients, but are not evident without (functional) genomic or isotopic data. Based on genomic approaches, for example, the cryptic cycling of sulfur species appears to be a common phenomenon in aqueous sediments (2, 9, 10). Sulfate is partially or fully reduced to sulfide by sulfate-reducing bacteria and then reoxidized under anaerobic or microaerobic conditions by autotrophic sulfur-oxidizing bacteria.

Combining genomics with transcriptomics or proteomics enables metabolic activity to be linked to specific organisms within communities to determine individual contributions (Fig. 1). This enables us to better understand changes in the transcriptional or translational activities of specific taxa by taking into account structural shifts in communities, such as occur before and after an environmental perturbation. An important characteristic of microbial communities is that they are structurally dynamic, which in turn impacts how associated functional data can be interpreted. For example, a doubling of transcripts for a particular gene (based on relative abundance in the metatranscriptome), corresponding to an equivalent increase of the source organism, indicates that expression of the genes per cell remained constant across conditions. This is evident only when one considers changes in genome abundance. By correcting for changes in genome abundance using “taxon-specific scaling” of transcriptome count data (11), increases or decreases in gene expression can be elucidated (e.g., reference 9).

## MULTISCALED APPROACHES TO CAPTURE RESOLUTION AND SCALE

Currently, a major impediment to understanding ecosystem function is the cost for high-resolution analyses (for sequencing and in analysis time), which limits the scale of application. This is particularly problematic in environments where large sample numbers are needed to understand trends, due to a high level of spatial heterogeneity (e.g., soils) (4) or temporal dynamics (12). A multiscaled approach can be an effective way to overcome this problem, that is, by analyzing a wide set of samples at low resolution and smaller subsets at progressively higher resolutions (e.g., rRNA gene amplicons → inferred genomes → shallow metagenomes → deep metagenomes with MAGs) (Fig. 1). Such multiscaled approaches can be useful in demonstrating the environmental prevalence of key taxa, while also elucidating their lifestyles (e.g., genome reduction in abundant and ubiquitous “*Candidatus* Udaeobacter copiosus” in grassland soils) (4). The home microbiome study provides another illustration of this approach. Low-resolution community compositional analysis was used to survey homes for surfaces that share highly similar communities with their occupants, indicating surfaces frequently or recently touched (12). To corroborate this, communities on select surfaces were also shown to share identical microbial genomes with home occupants.

## BOUNDLESS OMICS: TRANSCENDING KINGDOM BARRIERS

The constraints of traditional marker gene assays, which segregate and ring-fence taxonomic groups of organisms, is beginning to fall away with untargeted sequencing of environmental DNA. The boundless quality of shotgun sequencing leads to the intentional or accidental recovery of nontarget (e.g., nonprokaryotic) DNA from environmental samples. While this is overtly the case for host microbiome studies, it can be equally valid for other environments where eukaryote DNA is co-recovered, such as terrestrial soil, marine sediment, or stream biofilms (9). The co-recovery of unwanted extra DNA can be problematic in terms of perceived sequencing resource theft. However, it can also provide useful insights into the ecologically wider taxonomic setting by, for example, directly juxtapositioning microbial abundances alongside those of higher organisms. One simple way to achieve this is through co-recovery of (nearly) full-length rRNA and rRNA gene sequences from metatranscriptomic and metagenomic data. This approach has revealed that following the Deepwater Horizon oil spill, there was not only a bloom of hydrocarbon-degrading bacteria in heavily polluted deep-sea sediments but also a stark increase in bacterial dominance over archaea and eukaryotes (e.g., diatoms and nematodes) (9).

Environmental genomic approaches are aptly suited to recovering the small tidy population genomes of bacteria, archaea, and viruses. Nevertheless, a natural extension of the method is to microbial eukaryotes: diverse protists, such as diatoms and ciliates (13), and also multicellular eukaryotes (14). The reconstruction of microbial eukaryote genomes from the environment has the potential to generate novel insights into the functional characteristics of these, in many cases enigmatic, organisms. Protist genome sizes, for example, can dwarf those of bacteria, making their assembly from environmental data challenging. However, some are not appreciably larger (e.g., typical diatom genome sizes are only tens of megabases), and large genome fragments are relatively easy to assemble from metagenomic data. Diverse multicellular eukaryote genomes are also recoverable from metagenomes (14). Importantly, these genomes can be combined with functional methods. Recently, my colleagues and I have used metaproteogenomics to compare bacterial and eukaryotic roles associated with ephemeral stream eutrophication. During a nutrient influx event, photosynthesizing cyanobacteria establish thick biofilms that are cohabited by diatoms; both biofilm architect and tenant contribute to stream primary production and eutrophication (unpublished data) (Fig. 1). Functional genomic data afford us the unprecedented opportunity to readily integrate microbial eukaryotes into environment-specific ecological frameworks alongside co-occurring bacteria and archaea. Despite the importance of protist morphological and behavioral traits (13), genomics can still provide valuable insights into the ecological roles of these organisms and bring the environmental contributions of protists into sharper focus for the wider microbial ecology research community.

## A FUTURE THAT IS MORE THAN MICROBIAL

Microbial ecologists may increasingly find themselves meeting with unexpected companions in the environmental molecular space. Environmental DNA (eDNA) is gaining popularity among diverse ecologists who research groups of organisms as wide ranging as fungi, metazoans, and plants. The appeal is due to the persistence of DNA in the environment and the strong potential of recovering the DNA of larger organisms from environmental sources, such as soil or filtered water, without needing to capture or physically isolate these organisms (15). The scope of eDNA is therefore broad and includes the DNA of decaying or shedding organisms (e.g., fish), as well as whole organisms (e.g., insects or microbial eukaryotes and prokaryotes). While much of the focus of this type of research is currently on marker gene-based taxonomic characterization (due to the inherent complexities of eukaryotic genomes), metagenomics holds promise for primer bias-free taxonomic (9), as well as functional-gene, assessments.
